# Effects of *Dictyophora* polysaccharide on cerebellar Purkinje cell degeneration in a chronic alcohol mouse model

**DOI:** 10.1002/ame2.70021

**Published:** 2025-04-13

**Authors:** Jian Zhang, Zhihui Dai, Huanhuan Yu, Baofei Sun, Jiuyang Ding, Yuanhe Wang

**Affiliations:** ^1^ School of Forensic Medicine Guizhou Medical University Guiyang China; ^2^ State Key Laboratory of Ore Deposit Geochemistry Institute of Geochemistry, Chinese Academy of Sciences Guiyang China; ^3^ Key Laboratory of Macrocyclic and Supramolecular Chemistry of Guizhou Province Guizhou University Guiyang China; ^4^ Key Laboratory of Human Brain Bank for Functions and Diseases of Department of Education of Guizhou Province Guizhou Medical University Guiyang China

**Keywords:** alcohol, cerebellum, *Dictyophora* polysaccharides, inflammasome, motor deficit

## Abstract

**Background:**

Recent research showed that the NLRP3 inflammasome was activated in the central nervous system of mice administered chronic ethanol (EtOH). *Dictyophora* polysaccharides (DIPs) are essential components of the valuable edible fungus *Dictyophora*, which has antioxidant properties that can delay the aging process of the body. This study aimed to investigate the roles of NLRP3 in chronic EtOH‐induced cerebellar Purkinje cell (PC) degeneration and behavioral changes.

**Methods:**

C57BL/6J normal and NLRP3 knockout mice were exposed to EtOH for 14 days. *Dictyophora* polysaccharide (DIP) and NLRP3 inhibitor were administered to the EtOH mice. The pathology and NLRP3‐ASC‐caspase‐1 signaling pathway proteins were analyzed in EtOH mice cerebellar tissues and behavioral performance was assessed in the mice.

**Results:**

In the EtOH mouse model, we observed increases in the NLRP3 inflammasome proteins, including NLRP3, ASC, caspase‐1, mature IL‐1β and pro IL‐1β, loss of PCs, and motor coordination disorders. We found that DIPs could suppress the NLRP3‐ASC‐caspase‐1 signaling pathway, and alleviate the motor deficits and cerebellar pathological changes in chronic EtOH mice. Next, we used MCC950, a NLRP3 inhibitor, and an NLRP3 knockout strategy to further verify the effects of NLRP3‐ASC‐caspase‐1 signaling in chronic EtOH mice. MCC950 or NLRP3 knockout alleviated the EtOH‐induced latency to decreases in fall time, increases in stride width and decreases in stride length. MCC950 or NLRP3 knockout also attenuated PC number loss and suppressed NLRP3 inflammation induced by EtOH. Taken together, pharmacologically or genetically inhibiting NLRP3 alleviated EtOH‐induced cerebellar degeneration and behavioral deficits.

**Conclusion:**

These findings indicated that DIPs might diminish EtOH‐induced cerebellar degeneration and behavioral deficits through the NLRP3‐ASC‐caspase‐1 signaling pathway, which provides a potential therapeutic target for the prevention and treatment of alcoholism and EtOH‐induced cerebellar pathology.

## Graphical abstract

In an ethanol (EtOH)‐treated mouse model, increases in the NLRP3 inflammasome proteins, PC degeneration, and motor coordination disorders were observed. DIP suppressed the NLRP3‐ASC‐caspase‐1 signaling pathway, and alleviated the motor deficits and cerebellar pathological changes in chronic EtOH‐treated mice. MCC950, a NLRP3 inhibitor, and an NLRP3 knockout strategy were used to further verify the effects of NLRP3‐ASC‐caspase‐1 signaling in chronic EtOH‐treated mice. Pharmacologically or genetically inhibiting NLRP3 alleviated EtOH‐induced cerebellar degeneration and behavioral deficits.

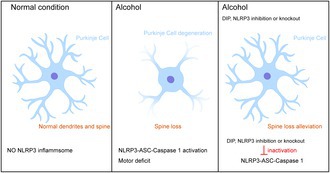

## 1 INTRODUCTION

Alcohol is a psychoactive substance, and heavy alcohol intake leads to different degrees of damage to various organs of the body, especially to the central nervous system, liver and heart,^1^, ^2^ which can seriously affect the quality of daily life.^3^ Previous studies have shown that 3 million people die from diseases or injuries related to alcohol abuse every year worldwide, accounting for 5.3% of all deaths. Most of the deaths attributed to alcohol occur particularly among the population aged 20–39 years.^4^ Heavy drinking has also been shown to lead to the occurrence of cancers, such as liver cancer, colon cancer, stomach cancer, oral cancer, etc.,^5^, ^6^, ^7^, ^8^ which undoubtedly place huge economic and psychological burdens on many families.

The inflammasome is composed of a nucleotide‐binding domain leucine‐rich repeat‐containing protein (NLRP3), an apoptosis‐associated speck‐like protein containing a CARD (ASC) and pro‐caspase‐1. Recent research showed that the NLRP3 inflammasome was activated in the central nervous system of chronic EtOH mice,^9^ while our previous study found that methamphetamine‐induced cerebellar degeneration was alleviated after treatment with the NLRP3 inhibitor MCC950.^10^ Another study also indicated that EtOH up‐regulated and activated the NLRP3 inflammasome, leading to caspase‐1 activation and increases in mature IL‐1β in the cerebellum. The IL‐1β‐triggered neuroinflammation could be blocked by disruption of IL‐1/IL‐1β signaling in the EtOH mouse model,^11^ and IL‐1β protein levels and caspase‐1 activity did not increase in EtOH‐fed NLRP3 knockout or ASC knockout mice compared with control mice. However, caspase‐1 activity and mature IL‐1β protein increased in EtOH‐fed wild type mice compared with EtOH‐fed NLRP3 knockout or ASC knockout mice. These results suggest that lack of NLRP3 or ASC could prevent the increase of IL‐1β induced by EtOH in the cerebrum.^11^

*Dictyophora* polysaccharides (DIPs) are an essential component of *Dictyophora*, a valuable edible fungus with antioxidant properties that can delay the aging process of the body.^12^ According to a recent research, DIPs have a therapeutic impact on rats that have been poisoned by arsenic.^13^ However, the effects of DIPs on EtOH‐induced cerebellar degeneration remained unknown.

In the central nervous system, the cerebellum is an important target organ of alcohol.^14^, ^15^ Therefore, we chose the cerebellum as the area of our research. The cerebellum is involved in control of balance and motor movement and thus we observed the motor performance in EtOH treated mice. To gain a thorough understanding of DIPs on EtOH‐induced cerebellar degeneration, we exposed EtOH treated mice to low and high doses of DIP. Moreover, MCC950, an NLRP3 inhibitor, and NLRP3 knockout mice were utilized to observe the effects of NLRP3 inhibition on chronic EtOH treated mice. Therefore, the study aimed to explore the effects of ethanol exposure on cerebellar PCs and the protective effect of DIP in a chronic EtOH mice model. We also aimed to discover whether the NLRP3 inflammatory pathway was involved in EtOH‐induced cerebellar PC degeneration. In addition, the study might provide a potential therapeutic target for the prevention and treatment of alcoholic brain injury.

## 2 METHODS

### 2.1 Animals

C57BL/6J mice (20–25 g, 8 weeks old) were purchased from the Laboratory Animal Center of Guizhou Medical University. The NLRP3 knockout mice (C57BL/6Smoc‐Nlrp3^em3Smoc^) were purchased from Shanghai Model Organisms (Catalog number NM‐KO‐190428). The mice were kept in cages (3 mice per cage) in the animal experiment room, in a clean, quiet environment, with free access to food and water. All animal experiments were approved by the Institutional Animal Care and Use Committee of Guizhou Medical University (Approval No. 2304547) and were performed according to *National Institutes of Health Guidelines*.

### 2.2 Chronic alcohol exposure and experimental groups

EtOH was administered (v/v 20%, 4 g/kg body weight) once a day^16^ and the doses of DIP were set at 25 mg/kg for the low dose and 100 mg/kg for high dose and administered once a day.

**FIGURE 1:**
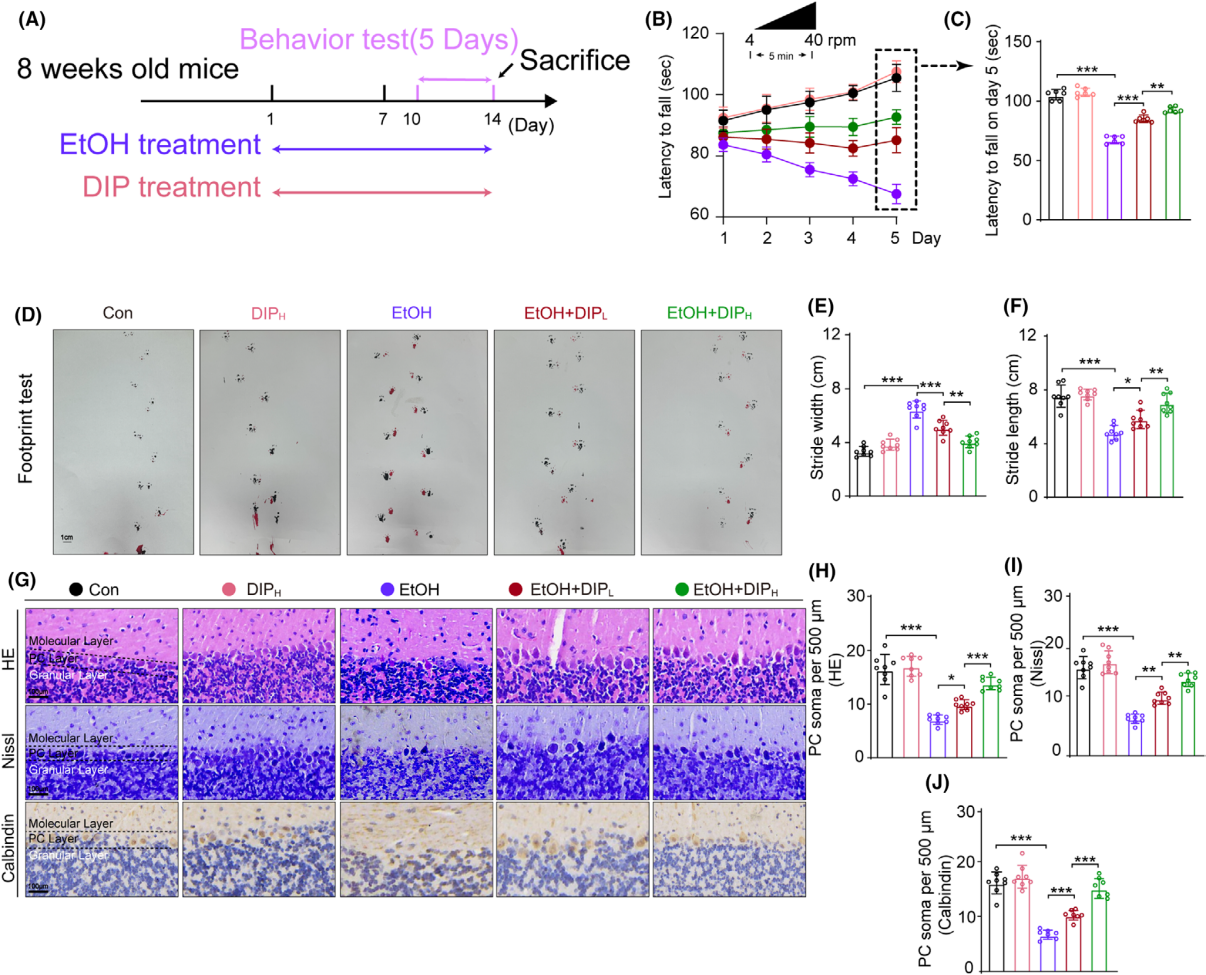
DIP alleviated PC number loss and behavioral abnormalities in a chronic EtOH mouse model. (A) Experimental design of the EtOH and DIP treatment regimen. (B) Rotarod test performance in each group mice from day 1 to day 5, *n* = 6. Repeated measures ANOVA, *F*_day×treatment_ (16, 149) = 72.256, *p* < 0.001; *F*_day_ (4, 149) = 29.568, *p* < 0.001; *F*_treatment_ (4, 149) = 125.547, *p* < 0.001. (C) Latency to fall on day 5 in rotarod test. One‐way ANOVA, *F* (4, 25) = 137.529, *p* < 0.001. (D) Effect of DIP on footprint pattern of mice treated with EtOH, *n* = 8. Bar = 1 cm. (E and F) Stride length and stride width analysis in footprint test. Stride width: *F* (4, 35) = 50.980, *p* < 0.001, Stride length: *F* (4, 35) = 50.980. (G) HE staining, Nissl staining and calbindin IHC staining in the cerebellum, Bar = 100 μm. (H–J) Comparison of PC soma number from HE staining, Nissl staining and calbindin IHC staining results. HE staining: *F* (4, 35) = 51.471, *p* < 0.001, Nissl staining: *F* (4, 35) = 60.178, *p* < 0.001, calbindin IHC staining: *F* (4, 35) = 65.435, *p* < 0.001. *n* = 8 per group by one‐way ANOVA and Bonferroni's post hoc analysis, *p < 0.05, **p < 0.01, ***p < 0.001.

In the first phase: the mice were randomly divided into five groups as follows:

1. Con: Saline was administered intraperitoneally to control group mice in place of 20% EtOH;
2. EtOH: 20% EtOH was administered intraperitoneally as shown in Figure 1A;
3. Con + DIP_H_ (100 mg/kg): Mice were injected intraperitoneally with DIP (100 mg/kg) once daily from day 1 to day 14 as shown in Figure 1A;
4. EtOH + DIP_L_ (25 mg/kg): Both EtOH and DIP (25 mg/kg) were injected intraperitoneally once daily from day 1 to day 14;
5. EtOH + DIP_H_ (100 mg/kg): Both EtOH and DIP (100 mg /kg) were injected intraperitoneally once daily from day 1 to day 14.

In the second phase, the mice were randomly divided into four groups:

1. Con: Saline was administered intraperitoneally to control group mice in place of 20% EtOH;
2. EtOH: 20% EtOH was administered intraperitoneally as shown in Figure 3A;
3. Con + MCC950 (20 mg/kg): Mice were intraperitoneally injected with MCC950 (20 mg/kg) once daily from day 1 to day 14 as shown in Figure 3A;
4. EtOH + MCC950 (20 mg/kg): Both EtOH and MCC950 (20 mg/kg, once daily) were intraperitoneally injected for a total of 14 days.

In the third phase, the mice were randomly divided into four groups:

1. WT: Saline was administered intraperitoneally to wild type mice in place of 20% EtOH once daily from day 1 to day 14 as shown in Figure 5A;
2. NLRP3‐KO: Saline was administered intraperitoneally to NLRP3 knockout mice in place of 20% EtOH once daily from day 1 to day 14 as shown in Figure 5A.
3. WT + EtOH: 20% EtOH was administered intraperitoneally to wild type mice as shown in Figure 5A;
4. NLRP3‐KO + EtOH: 20% EtOH was administered intraperitoneally to NLRP3 knockout mice as shown in Figure 5A.

### 2.3 Footprint analysis

We conducted the gait test according to the method we used previously.^10^ The aim of footprint testing is to evaluate the coordination of mouse movements.^17^, ^18^ The testing tool is a wooden runway that is U‐shaped and has a length of 40 cm and a width of 10 cm. A piece of white A4 paper (210 mm × 297 mm) is positioned at the bottom of the wooden board. The front and hind paws of the mice were painted, respectively, red and black prior to the test. Before the formal test, the mice were trained for 3 days. The mice were free to move on the runway during the test. The mice footprints were captured using a camera. Stride length was measured as the average distance between each forepaw and hindpaw footprint. Stride width was measured as the average distance between the right and left footprint of each forepaw and hindpaw. These measurements were analyzed across all groups.

### 2.4 Rotarod test

The rotarod test evaluated the balance and motor coordination of the mice.^17^, ^19^ In this test, mice balance on a rod rotating at speeds accelerating between 4 and 40 rpm. Over the course of 5 days, the test lasted 5 min each day, during which animals were trained for the first 3 days. The average latency to fall is recorded for each animal after three tests.

### 2.5 Western blot (WB)

The cerebellar tissues of each group of mice were collected and stored in liquid nitrogen. After crushing with a mortar, the tissues were carefully collected and a proteinase inhibitor was added. The tissues were centrifuged at 1300 rpm at 4°C for 15 min and the resulting supernatant was added to EP tubes. The BCA protein quantification method was utilized to measure the protein concentration of every sample, and the average concentration was obtained by measuring each sample three times. The samples were separated by SDS‐PAGE at a ratio of 5:1 after shaking to distribute the contents evenly. Targeted proteins were transferred onto PVDF membranes (Millipore, MA, USA). After blocking in 5% skim milk for 2 h, the membranes were incubated at 4°C overnight with primary antibodies, including rabbit monoclonal NLRP3 antibody (Cat#ab4207, diluted 1:1000, Abcam, USA), rabbit monoclonal ASC antibody (Cat#ab151700, diluted 1:5000, Abcam, USA), rabbit monoclonal cleaved caspase‐1 antibody (Cat#89332, diluted 1:2000, CST technology, USA), rabbit monoclonal caspase‐1 antibody (Cat#24232, diluted 1:2000, CST Technology, USA), mature‐IL‐1β antibody (Cat#A1112, diluted 1:2000, CST, China), and IL‐1β antibody (Cat#12703, diluted 1:2000, CST Technology, USA). Subsequently, the membranes were incubated with sufficient secondary antibodies: HRP‐conjugated goat anti‐mouse IgG antibody (ab6721, diluted 1:10000, CST Technology, USA) and HRP‐conjugated goat anti‐rabbit IgG antibody (ab6728, diluted 1:10000, CST Technology, USA). Chemiluminescent reagents were utilized to visualize the blots. GAPDH was used to normalize all protein expression levels.

### 2.6 Hematoxylin–Eosin (HE) staining

For HE staining, the mouse cerebellar tissues were collected and placed in 4% PFA for fixation for 24 h, and then dehydration and paraffin embedding were carried out. Sections 3 μm thick were cut using a microtome (RM 2235, Leica, Germany). Hematoxylin stain was added for 1 min and Eosin stain was added for 30 s. The sections were then dehydrated using an ethanol gradient, cleared for transparency using xylene, and sealed using neutral gum. A microscope (RM 2235, Leica, Germany) was used for observation and photography.

### 2.7 Immunohistochemistry (IHC) staining and Nissl staining

For IHC staining, the 4% PFA fixed tissues were cut into 5 μm sections using a microtome (RM 2235, Leica, Germany). After antigen retrieval and blocking, the sections were incubated with the rabbit monoclonal calbindin antibody (Cat#ab108404, diluted 1:300, Abcam, USA) overnight at 4°C. The target protein was visualized using a 3,3’‐Diaminobenzidine (DAB) kit (CW 2069, CWBio, China). A microscope (CX23, Olympus, Japan) was employed to capture images.

For Nissl staining, the cerebellar sections were incubated with Nissl Staining Buffer (C0117, Beyotime) for 10 min. Next, the slices were rapidly rinsed in ddH_2_O before dehydration. Images were obtained using a microscope (CX23, Olympus, Japan).

### 2.8 Transmission electron microscope analysis (TEM)

The TEM samples of mice brain tissues were performed as described in our previous studies.^20^ Briefly, cerebellum tissues were fixed in 2.5% glutaraldehyde at 4°C for 12 h, and embedded in Epon resin (Polyscience, Inc. Eppelheim, Germany) at 65°C for 2 days. Then the tissues were sectioned (7 nm in thickness) using an ultramicrotome (EM UC7, Leica, Wetzlar, Germany). The ultrathin sections were stained with lead citrate before imaging using an electron microscope (Tecnai G2, FEI, CA, USA).

### 2.9 Statistical analysis

All data were expressed mean ± SEM and were analyzed using SPSS 22.0. A one‐way ANOVA with Bonferroni's post hoc or repeated measures ANOVA test was conducted for statistical analysis. Randomization and blind analyses were used in the behavioral tests and pathological analysis. Statistical significance was set at *p* < 0.05. The number of different experimental groups is reported in the figure legends.

## 3 RESULTS

### 3.1 DIP improved motor coordination performance and PC degeneration in mice subjected to chronic EtOH exposure

The EtOH mice exhibited a decrease in the latency to fall compared with that of control mice in the rotarod test. In the EtOH + DIP groups, on the other hand, the latency increased, with the high‐concentration DIP (100 mg/kg) having a particularly obvious effect. On the 5th day, mice given 100 mg/kg DIP with EtOH had a similar latency to fall to the control mice (Figure 1B,C). The EtOH mice showed shorter stride lengths and wider stride widths compared with the control mice, while the low‐ and high‐dose EtOH + DIP mice showed longer stride lengths and a narrower stride widths compared with EtOH mice, with the high‐dose DIP group mice exhibiting greater improvement (Figure 1D–F).

The number of PC cell bodies was observed using HE staining, Nissl staining, and calbindin (a marker of PCs^21^) IHC staining (Figure 1G). The number of PCs was decreased in EtOH group mice compared with control group mice, and this could be alleviated by administering 20 or 100 mg/kg of DIP (Figure 1G–J).

### 3.2 DIP decreased IL‐1β secretion through the NLRP3‐ASC‐caspase‐1 pathway in our EtOH mice model

**FIGURE 2:**
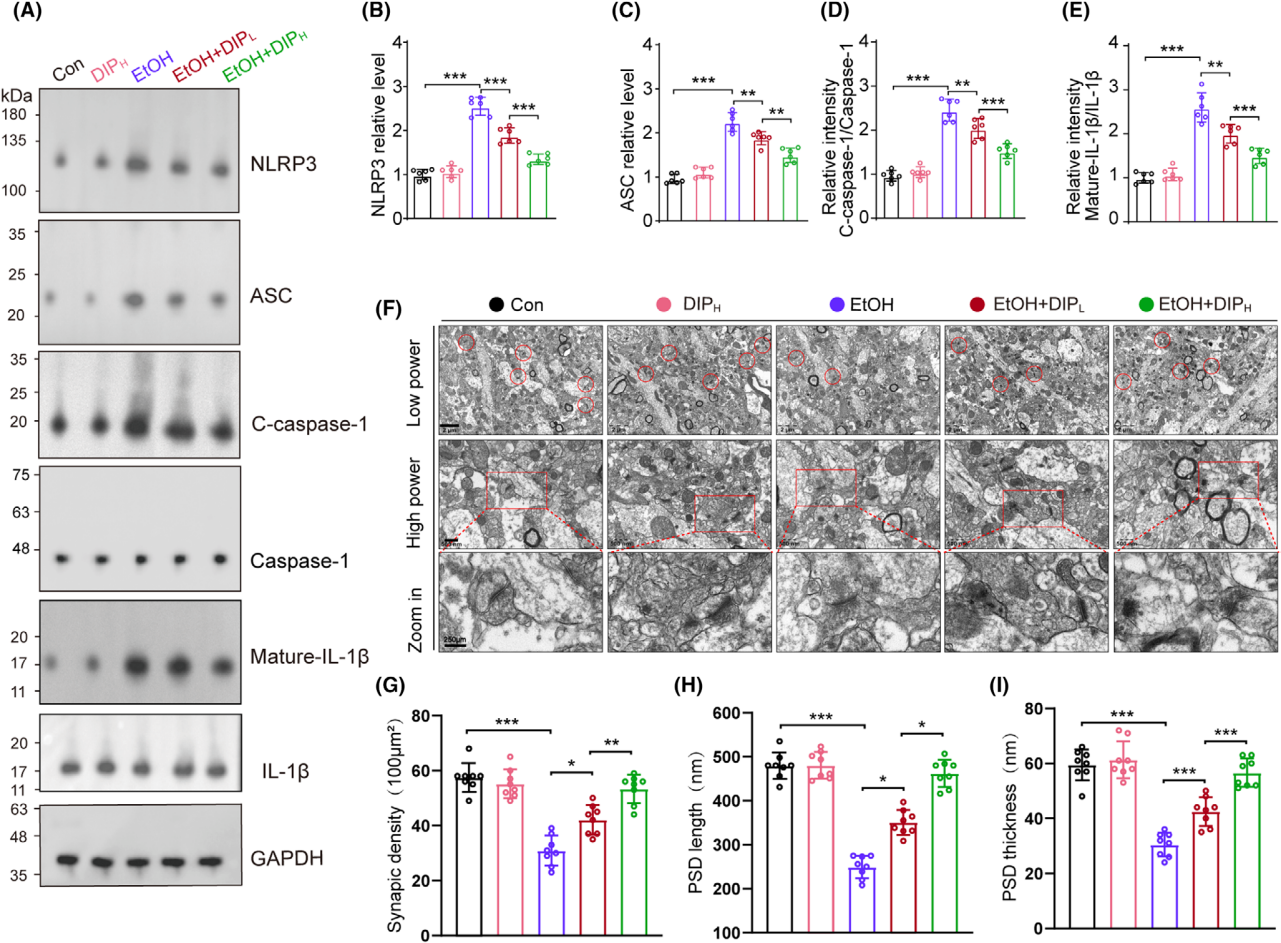
DIP suppressed NLRP3‐ASC‐caspase‐1 activation and synaptic degeneration induced by EtOH. (A) Western blot for NLRP3, ASC, caspase‐1, C‐caspase‐1, mature‐IL‐1β, IL‐1β and GAPDH in cerebellum. *n* = 6. (B–E) Relative intensity of NLRP3, ASC, C‐caspase‐1 and mature‐IL‐1β in cerebellar areas. NLRP3 relative level: One‐way ANOVA, *F* (4, 25) = 105.596, *p* < 0.001; ASC relative level: *F* (4, 25) = 71.285, *p* < 0.001; Relative intensity C‐caspase‐1/caspase‐1: *F* (4, 25) = 71.828, *p* < 0.001; Relative intensity mature IL‐1β/IL‐1β: *F* (4, 25) = 62.571, *p* < 0.001. (F) Representative electron micrographs of synapses in cerebellum of mice in Con, DIP_H_, EtOH, EtOH + DIP_L_, and EtOH + DIP_H_ groups. Red circles indicate synapses. (G) Quantification of synaptic density in cerebellum. One‐way ANOVA, *F* (4, 35) = 46.5, *p* < 0.001. (H) Quantification of PSD length in cerebellum. One‐way ANOVA, *F* (4, 35) = 97.19, *p* < 0.001. (I) Quantification of PSD thickness in cerebellum. One‐way ANOVA, *F* (4, 35) = 35.48, *p* < 0.001. *n* = 8 per group by one‐way ANOVA and Bonferroni's post hoc analysis, *p < 0.05, **p < 0.01, ***p < 0.001.

In order to explore the mechanism of the protective effect of DIP on EtOH‐induced cerebellar degeneration, we analyzed the NLRP3 pathway proteins. We found that NLRP3, ASC, cleaved caspase‐1 and mature IL‐1β proteins were increased in the cerebella of EtOH‐treated mice. In addition, DIP (20 mg/kg, 100 mg/kg) inhibited the levels of increase of NLRP3, cleaved caspase‐1 and mature IL‐1β proteins induced by EtOH. The EtOH + 100 mg/kg DIP treatment was more significantly inhibitory than EtOH + 20 mg/kg DIP treatment (Figure 2A–E).

### 3.3 DIP alleviated synaptic degeneration induced by EtOH

Next, we observed the synaptic morphology in cerebellum. Compared with the control mice, EtOH induced a significant decrease in synaptic number and (postsynaptic density) PSD length and thickness were significantly reduced. Both low and high doses of DIP alleviated the reduction in synaptic numbers, PSD length and PSD thickness compared with that in EtOH mice (Figure 2F–I).

### 3.4 Administration of MCC950 protected against PC loss and behavioral changes in our EtOH mice model

**FIGURE 3:**
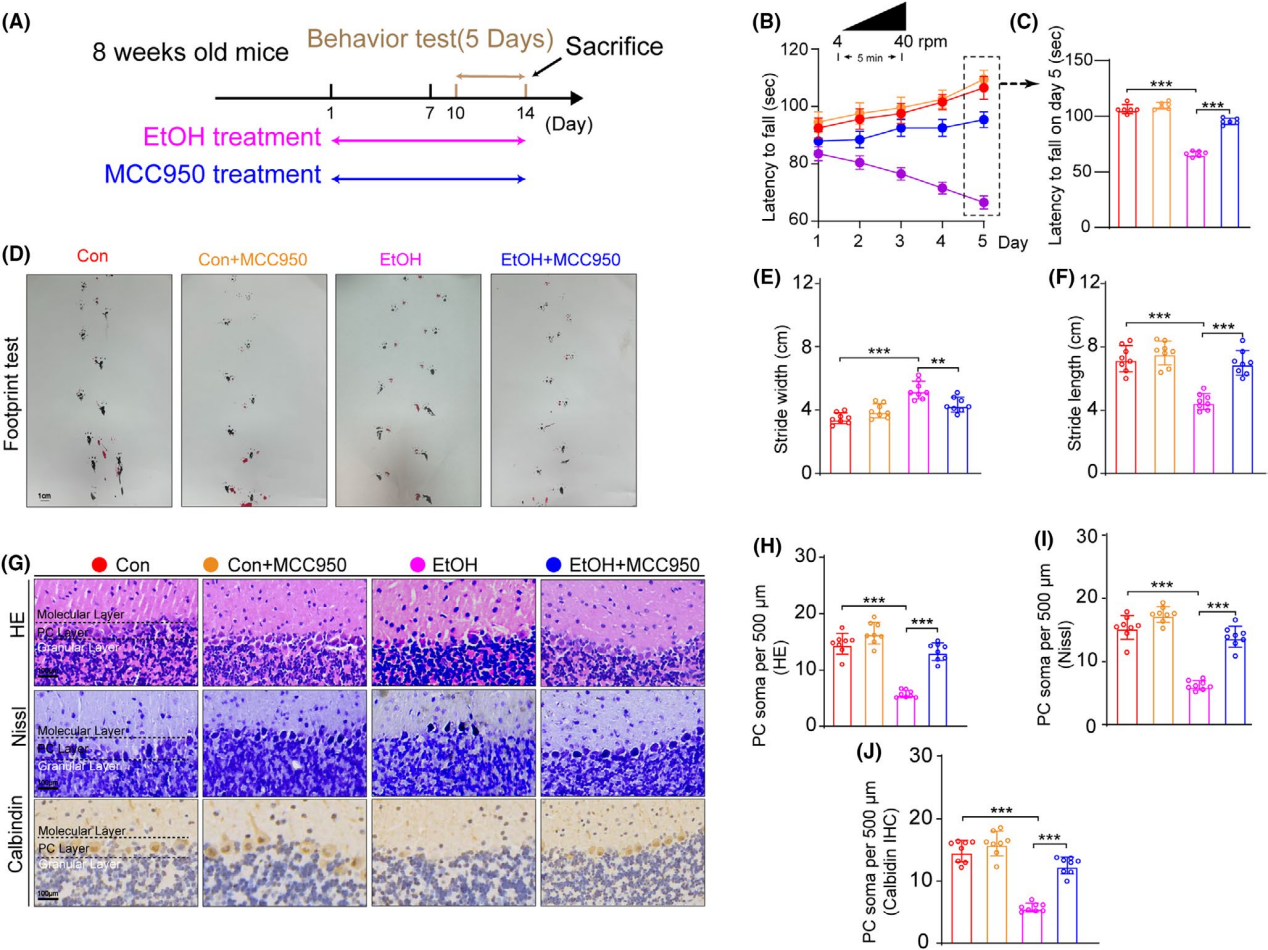
MCC950 alleviated PC number loss and behavioral abnormalities in a chronic EtOH mouse model. (A) Experimental design of EtOH and MCC950 treatment regimen. (B) Rotarod test performance in each group of mice from day 1 to day 5, *n* = 6. *F*_day×treatment_ (16, 149) = 102.247, *p* < 0.001; *F*_day_ (4, 149) = 49.589, *p* < 0.001; *F*_treatment_ (4, 149) = 156.587, *p* < 0.001. (C) Latency to fall on day 5 in rotarod test. One‐way ANOVA, *F* (3, 20) = 235.743, *p* < 0.001. (D) Effect of MCC950 on footprint pattern of mice treated with EtOH, *n* = 8. Bar = 1 cm. (E and F) Stride length and Stride width analysis in footprint test. Stride width: *F* (3, 28) = 21.613, *p* < 0.001; stride length: *F* (3, 28) = 29.472, *p* < 0.001. (G) HE staining, Nissl staining and calbindin IHC staining in the cerebellar area. Bar = 100 μm. (H–J) Comparison of PC soma number from HE staining, Nissl staining and calbindin IHC staining results. HE staining: *F* (3, 28) = 71.877, *p* < 0.001, Nissl staining: *F* (3, 28) = 92.113, *p* < 0.001, calbindin IHC staining: *F* (3, 28) = 73.688, *p* < 0.001. *n* = 8 per group by one‐way ANOVA Bonferroni's post hoc analysis, *p < 0.05, **p < 0.01, ***p < 0.001.

Compared with the control group mice, EtOH significantly reduced the latency to fall in the rotarod test. When we administered the NLRP3 inhibitor MCC950 in our EtOH mouse model, however, the fall latency of EtOH‐induced mice improved (Figure 3B,C). EtOH‐treated mice had a shorter stride length and a wider stride width during the gait test compared to the control mice, which was reversed by MCC950 intervention (Figure 3D–F).

The number of PC soma in the EtOH mice group was lower than in the control group. Administration of MCC950 (20 mg/kg) alleviated the loss of PC soma number caused by EtOH, and we noted no significant difference in PC numbers between the Con + MCC950 (20 mg/kg) group and control mice (Figure 3G–J).

### 3.5 MCC950 suppressed the activation of NLRP3 inflammasome signaling and alleviated synaptic degeneration

**FIGURE 4:**
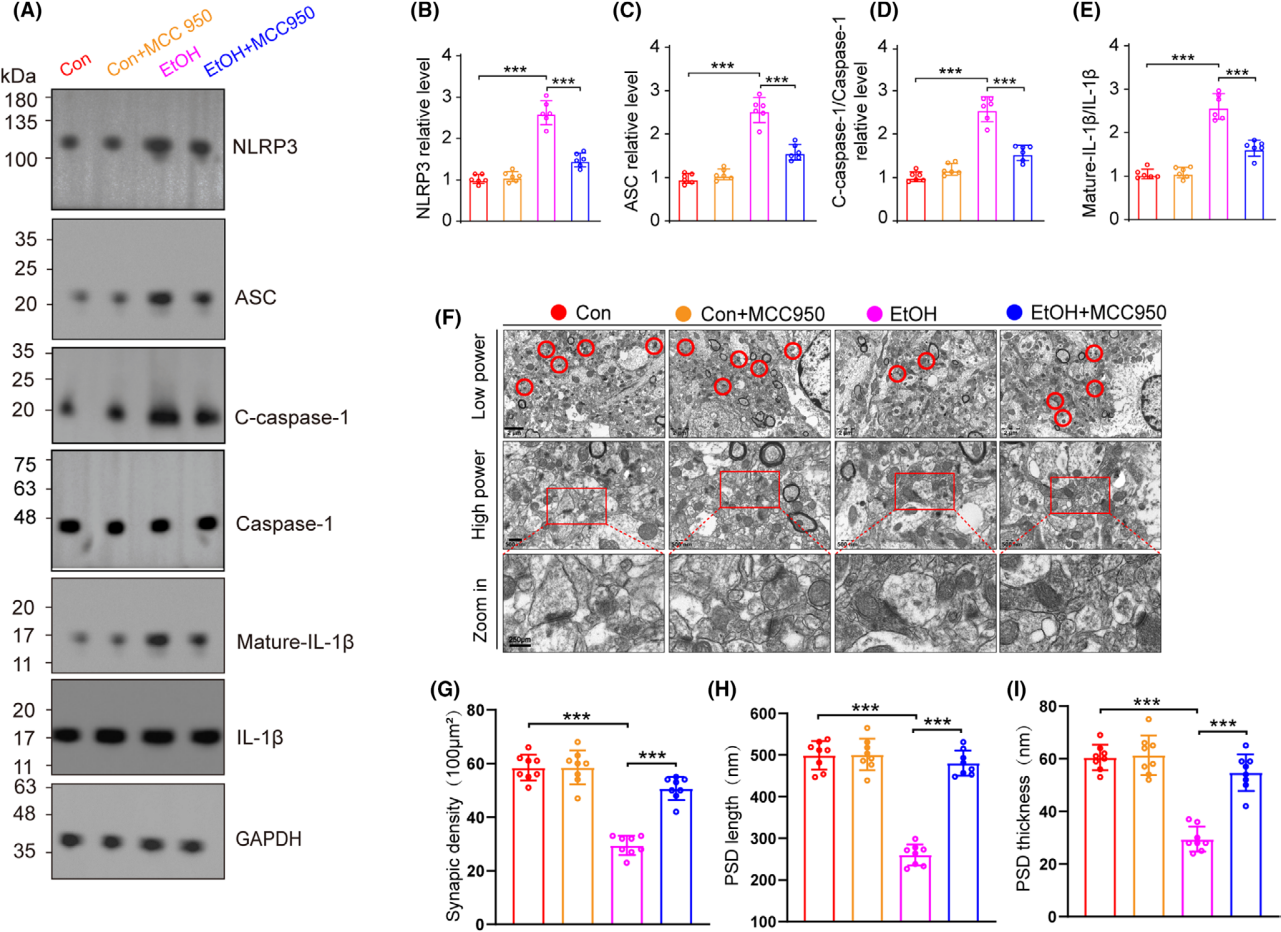
NLRP3 inhibition alleviated synaptic degeneration in chronic EtOH mouse model. (A) Western blot of NLRP3, ASC, caspase‐1, C‐caspase‐1, mature IL‐1β and IL‐1β in cerebellum, *n* = 6. (B–E) Relative intensity of NLRP3, ASC, C‐caspase‐1 and mature‐IL‐1β in cerebellar tissues. NLRP3 relative level: One‐way ANOVA, *F* (3, 20) = 94.171, *p* < 0.001; ASC relative level: *F* (3, 20) = 86.809, *p* < 0.001; Relative intensity C‐caspase‐1/caspase‐1: *F* (3, 20) = 80.605, *p* < 0.001; Relative intensity mature‐IL‐1β/IL‐1β: *F* (3, 20) = 85.362, *p* < 0.001. (F) Representative electron micrographs of synapses in cerebellum of mice in Con, Con + MCC950, EtOH and EtOH + MCC950 groups. Red circles indicate synapses. (G) Quantification of synaptic density in cerebellum. One‐way ANOVA, *F* (3, 28) = 63.87, *p* < 0.001. (H) Quantification of PSD length in cerebellum. One‐way ANOVA, *F* (3, 28) = 104.6, *p* < 0.001. (I) Quantification of PSD thickness in cerebellum. One‐way ANOVA, *F* (3, 28) = 47.35, *p* < 0.001. *n* = 8 per group by one‐way ANOVA and Bonferroni's post hoc analysis, *p < 0.05, **p < 0.01, ***p < 0.001.

Next, we investigated the effect of MCC950 on NLRP3 and its downstream pathway ASC/caspase‐1/C‐caspase‐1 (P20)/IL‐1β proteins. We found that the increased levels of NLRP3, ASC, C‐caspase‐1 and mature IL‐1β induced by EtOH were diminished by MCC950 (Figure 4A–E). Moreover, we found that MCC950 alleviated the *synaptic degeneration* induced by EtOH (Figure 4F–I).

### 3.6 Motor coordination disorders and increased PC number loss caused by chronic EtOH treatment were alleviated in NLRP3 knockout mice

**FIGURE 5:**
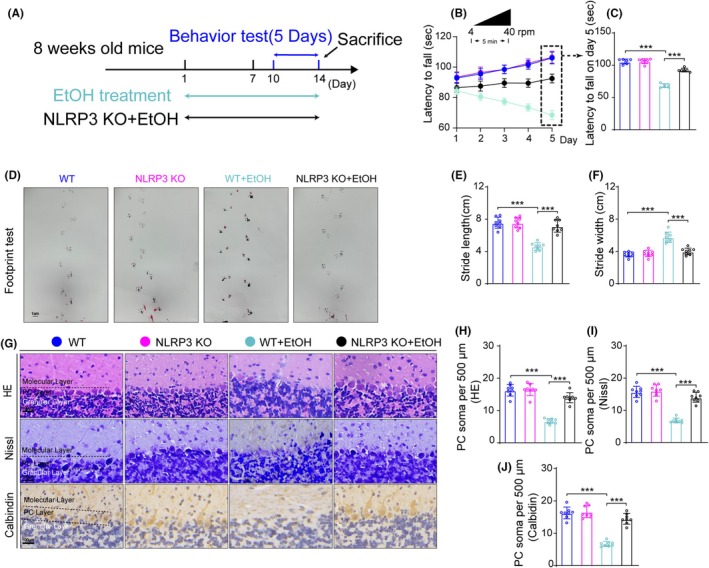
NLRP3 knockout ameliorated motor coordination impairment and alleviated loss of PC numbers in chronic EtOH mouse model. (A) Experimental design of EtOH and NLRP3 knockout treatment regimen. (B) Rotarod test performance in each group mice from day 1 to day 5, *n* = 6. *F*_day×treatment_ (16, 149) = 130.211, *p* < 0.001; *F*_day_ (4, 149) = 72.596, *p* < 0.001; *F*_treatment_ (4, 149) = 152.214, *p* < 0.001. (C) Latency to fall on day 5 in rotarod test. One‐way ANOVA, *F* (3, 20) = 160.355, *p* < 0.001. (D) Effect of NLRP3 knockout on footprint pattern of mice treated with EtOH, *n* = 8. Bar = 1 cm. (E and F) Stride length and stride width analysis in footprint test. Stride width: *F* (3, 28) = 37.059, *p* < 0.001; stride length: *F* (3, 28) = 37.701, *p* < 0.001. (G) HE staining, Nissl staining and calbindin IHC staining in the cerebellar area. Bar = 100 μm. (H–J) Comparison of PC soma number from HE staining, Nissl staining and calbindin IHC staining results. HE staining: *F* (3, 28) = 69.257, *p* < 0.001; Nissl staining: *F* (3, 28) = 66.473, *p* < 0.001; calbindin IHC staining: *F* (3, 28) = 70.165, *p* < 0.001. *n* = 8 per group by one‐way ANOVA and Bonferroni's post hoc analysis, *p < 0.05, **p < 0.01, ***p < 0.001.

Compared with the control group mice, mice treated with EtOH had a significantly reduced latency to fall in the rotarod test. However, knocking out NLRP3 could reverse the latency reduction (Figure 5B). In NLRP3 knockout mice, the fall latency in mice treated with EtOH was normalized when compared to control mice on day 5 (Figure 5C). EtOH‐treated mice had a shorter stride length and a wider stride width during the gait test compared to the control mice. In contrast, NLRP3 knockout could reverse the effect of EtOH (Figure 5D–F).

We then examined the effect of NLRP3 knockout on PC degeneration induced by EtOH. The number of PCs was observed by HE staining, Nissl staining and calbindin IHC staining. The number of PCs in the WT + EtOH group was reduced compared to the control group. NLRP3 knockout alleviated the reduction in PC numbers in EtOH mice (Figure 5G–J).

### 3.7 NLRP3 knockout mice showed suppressed activation of NLRP3 inflammasome signaling and less synaptic degeneration

**FIGURE 6:**
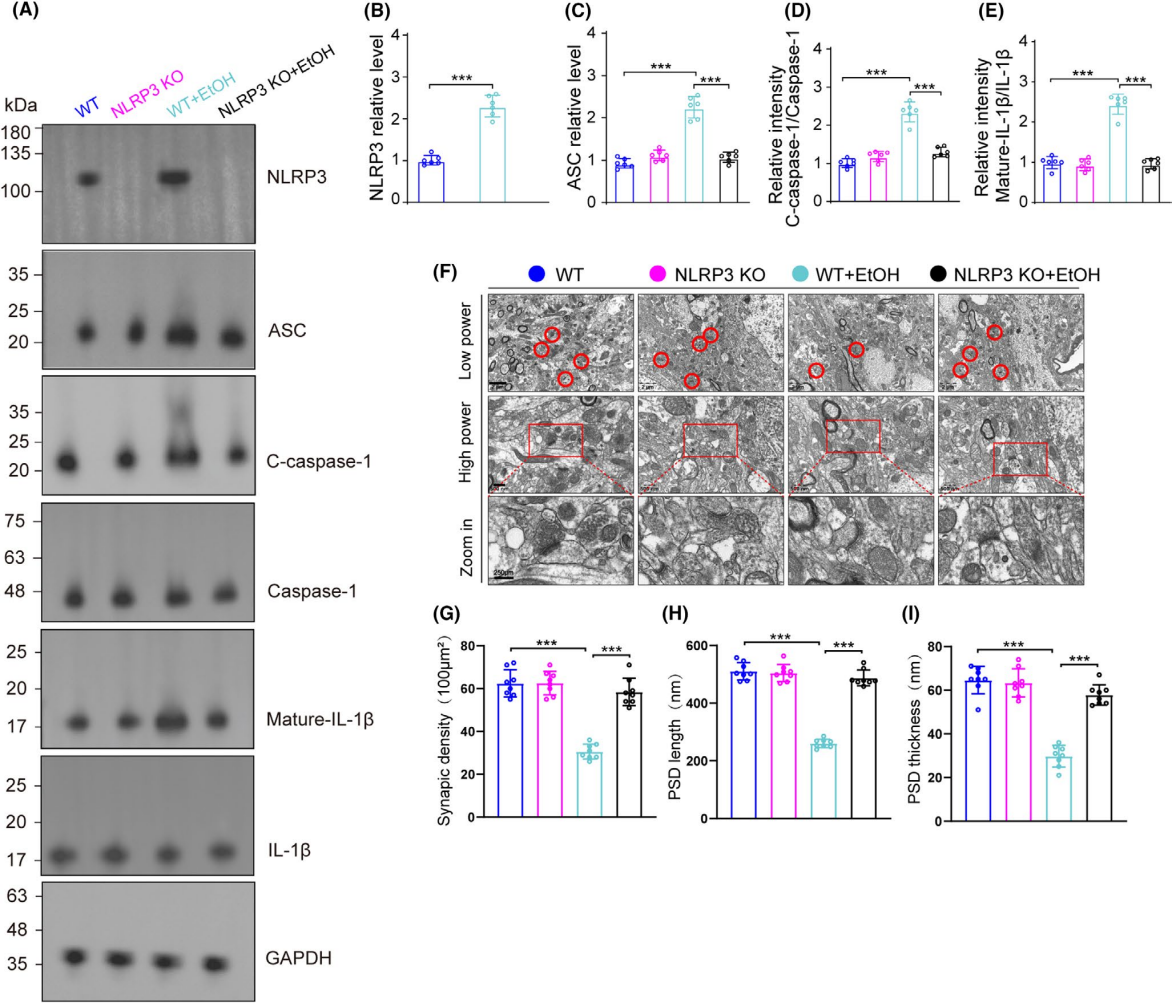
NLRP3 knockout alleviated synaptic degeneration in a chronic EtOH mouse model. (A) Western blot for NLRP3, ASC, caspase‐1, C‐caspase‐1, mature‐IL‐1β and IL‐1β in cerebellum, *n* = 6. (B–E) Relative intensity of NLRP3, ASC, C‐caspase‐1 and mature‐IL‐1β in cerebellar areas. NLRP3 relative level: One‐way ANOVA, *F* (3, 20) = 368.359, *p* < 0.001; ASC relative level: *F* (3, 20) = 83.106, *p* < 0.001; Relative intensity C‐caspase‐1/caspase‐1: *F* (3, 20) = 77.263, *p* < 0.001; Relative intensity mature‐IL‐1β/IL‐1β: *F* (3, 20) = 24.723, *p* < 0.001. (F) Representative electron micrographs of synapses in cerebellum of mice in WT, NLRP3 knockout, WT + EtOH and NLRP3‐KO + EtOH groups. Red circles indicate synapses. (G) Quantification of synaptic density in cerebellum. One‐way ANOVA, *F* (3, 28) = 61.88, *p* < 0.001. (H) Quantification of PSD length in cerebellum. One‐way ANOVA, *F* (3, 28) = 167.8, *p* < 0.001. (I) Quantification of PSD thickness in cerebellum. One‐way ANOVA, *F* (3, 28) = 67.73, *p* < 0.001. *n* = 8 per group by one‐way ANOVA and Bonferroni's post hoc analysis, *p < 0.05, **p < 0.01, ***p < 0.001.

We investigated the effect of NLRP3 knockout on the ASC/caspase‐1/C‐caspase‐1 (P20)/IL‐1β signaling pathway proteins (Figure 6A–E). We found the increased levels of NLRP3, ASC, C‐caspase‐1and Mature IL‐1β induced by EtOH were diminished by NLRP3 knockout mice. Moreover, we found that the NLRP3 knockout mice also alleviated the *synaptic degeneration* induced by EtOH (Figure 6F–I).

## 4 DISCUSSION

In our study, we found that EtOH impaired motor ability and caused neuropathological changes including cerebellar PC number loss and synaptic degeneration, and the NLRP3‐ASC‐caspase‐1 signaling pathway was activated in the EtOH mouse model. DIP, an essential component of bamboo fungus, could alleviate EtOH‐induced motor disability and neuropathology and reduce NLRP3‐ASC‐caspase‐1 signaling activation. Pharmacological or genetic modulation of NLRP3 diminished the EtOH‐induced motor disability and neuropathology in EtOH mice (note that the NLRP3‐ASC‐caspase‐1 signaling is activated during EtOH exposure). As far as we know, this is the first research to show that the modulation of NLRP3 by DIP has a protecting effect on the behavioral abnormalities and cerebellar degeneration of the mice treated with EtOH.

The NLRP3 inflammasome is activated in other organs under alcohol treatment, such as the liver, heart, lung, and kidney.^22^, ^23^, ^24^, ^25^, ^26^ We therefore speculated that the NLRP3 inflammasome might also be activated in the cerebellum. We found that the NLRP3‐ASC‐caspase‐1 signaling pathway was activated in the cerebellar tissues of our EtOH mouse model. We also found that PC numbers decreased and synaptic degeneration increased. Consistent with our results, a number of studies have demonstrated that NLRP3 is involved in the formation of inflammasomes in cerebellar inflammation caused by alcohol.^27^, ^28^ DIP has been shown to relieve the neurotoxicity caused by arsenic.^29^, ^30^ Thus, we hypothesized that DIP might relieve the cerebellar degeneration induced by EtOH. We treated EtOH mice with low or high doses of DIP, and found that DIP did prevent the reduction in PC numbers and synaptic degeneration caused by EtOH. The NLRP3‐ASC‐caspase‐1 signaling was suppressed by DIP.

The PC is a unique neuron; it is the only efferent neuron in the cerebellar cortex and is crucial to the function of the cerebellum.^31^, ^32^ Previous studies have demonstrated that the loss of PCs is sufficient to drive behavioral changes, such as impaired limb motor coordination.^32^ Cerebellar PC dysfunction or degeneration is often accompanied by cerebellar ataxia.^33^ In our study, we showed that the number of PCs in the EtOH group mice decreased, which was sufficient to drive motor coordination defects in the rotarod test and footprint analysis test.

In order to verify that the NLRP3 inflammasome pathway was involved in the EtOH‐induced cerebellar degeneration, we used the NLRP3 inhibitor MCC950 and NLRP3 knockout mice treated with EtOH. We found that genetic and pharmacologic manipulation of NRLP3 alleviated EtOH‐induced neuropathological changes and motor deficit.

The possible mechanisms associated with our findings include antioxidant effects and inhibition of NLRP3 inflammatory responses. More in‐depth research is needed to elucidate the mechanisms. Our future plan is to use additional concentrations of EtOH in smaller increments, in order to explore the relationship between EtOH concentration and the associated neuropathology.

## 5 CONCLUSION

In summary, our results demonstrate that NLRP3 may be a therapeutic target for treating alcohol abuse, and DIP may be a potential drug for treating EtOH‐induced cerebellar degeneration. Our results provide an important basis for further investigation of the damage to the cerebellum caused by chronic alcohol exposure and the interventional effect of DIP.

## AUTHOR CONTRIBUTIONS

**Jian Zhang:** Conceptualization; project administration; writing – original draft. **Zhihui Dai:** Data curation; project administration; software. **Huanhuan Yu:** Methodology; project administration. **Baofei Sun:** Funding acquisition; project administration. **Jiuyang Ding:** Funding acquisition; project administration. **Yuanhe Wang:** Conceptualization; writing – review and editing.

## FUNDING INFORMATION

This work was supported by National Natural Science Foundation of China (42077313), Guizhou Provincial 2020 Science and Technology Subsidies (No. GZ 2020SIG) (to ZHD), Research Foundation for Advanced Talents of Guizhou Medical University (Grant No. University Contract of Doctors J [2021] 014), Natural Science Foundation of Guizhou Medical University Incubation Program (Grant No. 20NSP084), Guizhou Provincial Science and Technology Projects (Grant No. Qian Science Foundation‐ZK [2023] General 328), Guizhou Provincial Education Department Young Scientific Talent (Grant No. Qianjiaoji [2024] 93) (to JYD).

## CONFLICT OF INTEREST STATEMENT

The authors declare that they have no conflict of interest.

## ETHICS STATEMENT

All animal experiments were conducted in accordance with the principles of laboratory animal care and performed in compliance with the Animal Experimental Ethical Committee of Guizhou Medical University (No.2304547).

## Data Availability

The raw data supporting the conclusions of this article will be made available by the authors, without undue reservation.

## References

[1] Rocco A , Compare D , Angrisani D , Sanduzzi Zamparelli M , Nardone G . Alcoholic disease: liver and beyond. World J Gastroenterol. 2014;20(40):14652‐14659. doi:10.3748/wjg.v20.i40.14652 25356028 PMC4209531

[2] Roerecke M , Rehm J . Alcohol consumption, drinking patterns, and ischemic heart disease: a narrative review of meta‐analyses and a systematic review and meta‐analysis of the impact of heavy drinking occasions on risk for moderate drinkers. BMC Med. 2014;12:182. doi:10.1186/s12916-014-0182-6 25567363 PMC4203905

[3] Mukherjee S . Alcoholism and its effects on the central nervous system. Curr Neurovasc Res. 2013;10(3):256‐262. doi:10.2174/15672026113109990004 23713737

[4] Egervari G , Siciliano CA , Whiteley EL , Ron D . Alcohol and the brain: from genes to circuits. Trends Neurosci. 2021;44(12):1004‐1015. doi:10.1016/j.tins.2021.09.006 34702580 PMC8616825

[5] Cohen SM , DePhilippis D , Deng Y , et al. Perspectives on contingency management for alcohol use and alcohol‐associated conditions among people in care with HIV. Alcohol Clin Exp Res (Hoboken). 2023;47(9):1783‐1797. doi:10.1111/acer.15159 37524371 PMC10828101

[6] Deng W , Jin L , Zhuo H , Vasiliou V , Zhang Y . Alcohol consumption and risk of stomach cancer: a meta‐analysis. Chem Biol Interact. 2021;336:109365. doi:10.1016/j.cbi.2021.109365 33412155

[7] Gonzalez‐Palacios S , Compan‐Gabucio LM , Torres‐Collado L , et al. The protective effect of dietary folate intake on gastric cancer is modified by alcohol consumption: a pooled analysis of the StoP consortium. Int J Cancer. 2024;155:1367‐1375. doi:10.1002/ijc.35004 38757245 PMC11326987

[8] Urvalek AM , Osei‐Sarfo K , Tang XH , Zhang T , Scognamiglio T , Gudas LJ . Identification of ethanol and 4‐Nitroquinoline‐1‐oxide induced epigenetic and oxidative stress markers during Oral cavity carcinogenesis. Alcohol Clin Exp Res. 2015;39(8):1360‐1372. doi:10.1111/acer.12772 26207766 PMC4597780

[9] Lin X , Wang H , Zou L , et al. The NRF2 activator RTA‐408 ameliorates chronic alcohol exposure‐induced cognitive impairment and NLRP3 inflammasome activation by modulating impaired mitophagy initiation. Free Radic Biol Med. 2024;220:15‐27. doi:10.1016/j.freeradbiomed.2024.04.236 38679301

[10] Ding J , Shen L , Ye Y , et al. Inflammasome inhibition prevents motor deficit and cerebellar degeneration induced by chronic methamphetamine administration. Front Mol Neurosci. 2022;15:861340. doi:10.3389/fnmol.2022.861340 35431795 PMC9010733

[11] Amin‐Esmaeili M , Farokhnia M , Susukida R , et al. Reduced drug use as an alternative valid outcome in individuals with stimulant use disorders: findings from 13 multisite randomized clinical trials. Addiction. 2024;119(5):833‐843. doi:10.1111/add.16409 38197836 PMC11009085

[12] Deng C , Fu H , Xu J , Shang J , Cheng Y . Physiochemical and biological properties of phosphorylated polysaccharides from Dictyophora indusiata. Int J Biol Macromol. 2015;72:894‐899. doi:10.1016/j.ijbiomac.2014.09.053 25316421

[13] Wang G , Zuo P , Ding K , et al. Intervention study of Dictyophora polysaccharides on arsenic‐induced liver fibrosis in SD rats. Biomed Res Int. 2022;2022:7509620. doi:10.1155/2022/7509620 35402611 PMC8986371

[14] Chatterton BJ , Nunes PT , Savage LM . The effect of chronic ethanol exposure and thiamine deficiency on myelin‐related genes in the cortex and the cerebellum. Alcohol Clin Exp Res. 2020;44(12):2481‐2493. doi:10.1111/acer.14484 33067870 PMC7725981

[15] Jung ME . Alcohol withdrawal and cerebellar mitochondria. Cerebellum. 2015;14(4):421‐437. doi:10.1007/s12311-014-0598-8 25195804

[16] Niedzwiedz‐Massey VM , Douglas JC , Rafferty T , Kane CJM , Drew PD . Ethanol effects on cerebellar myelination in a postnatal mouse model of fetal alcohol spectrum disorders. Alcohol. 2021;96:43‐53. doi:10.1016/j.alcohol.2021.07.003 34358666 PMC8578461

[17] Nobrega C , Nascimento‐Ferreira I , Onofre I , et al. Silencing mutant ataxin‐3 rescues motor deficits and neuropathology in Machado‐Joseph disease transgenic mice. PLoS One. 2013;8(1):e52396. doi:10.1371/journal.pone.0052396 23349684 PMC3551966

[18] Sugimoto H , Kawakami K . Low‐cost protocol of footprint analysis and hanging box test for mice applied the chronic restraint stress. J Vis Exp. 2019;143:23‐35. doi:10.3791/59027 30735178

[19] Deacon RM . Measuring motor coordination in mice. J Vis Exp. 2013;75:e2609. doi:10.3791/2609 PMC3724562 23748408

[20] Ding J , Hu S , Meng Y , et al. Alpha‐Synuclein deficiency ameliorates chronic methamphetamine induced neurodegeneration in mice. Toxicology. 2020;438:152461. doi:10.1016/j.tox.2020.152461 32278788

[21] Wallace K , Veerisetty S , Paul I , May W , Miguel‐Hidalgo JJ , Bennett W . Prenatal infection decreases calbindin, decreases Purkinje cell volume and density and produces long‐term motor deficits in Sprague‐Dawley rats. Dev Neurosci. 2010;32(4):302‐312. doi:10.1159/000319506 20948182 PMC3123740

[22] Al‐Kharashi L , Attia H , Alsaffi A , et al. Pentoxifylline and thiamine ameliorate rhabdomyolysis‐induced acute kidney injury in rats via suppressing TLR4/NF‐kappaB and NLRP‐3/caspase‐1/gasdermin mediated‐pyroptosis. Toxicol Appl Pharmacol. 2023;461:116387. doi:10.1016/j.taap.2023.116387 36690085

[23] de Carvalho Ribeiro M , Szabo G . Role of the Inflammasome in liver disease. Annu Rev Pathol. 2022;17:345‐365. doi:10.1146/annurev-pathmechdis-032521-102529 34752711 PMC10501045

[24] Liu J , Li X , Ding L , Li W , Niu X , Gao D . GRK2 participation in cardiac hypertrophy induced by isoproterenol through the regulation of Nrf2 signaling and the promotion of NLRP3 inflammasome and oxidative stress. Int Immunopharmacol. 2023;117:109957. doi:10.1016/j.intimp.2023.109957 37012864

[25] Solopov PA , Colunga Biancatelli RML , Catravas JD . Alcohol increases lung angiotensin‐converting enzyme 2 expression and exacerbates severe acute respiratory syndrome coronavirus 2 spike protein subunit 1‐induced acute lung injury in K18‐hACE2 transgenic mice. Am J Pathol. 2022;192(7):990‐1000. doi:10.1016/j.ajpath.2022.03.012 35483427 PMC9040477

[26] Yu C , Chen P , Miao L , Di G . The role of the NLRP3 Inflammasome and programmed cell death in acute liver injury. Int J Mol Sci. 2023;24(4):1‐20. doi:10.3390/ijms24043067 PMC9959699 36834481

[27] Munshi S , Albrechet‐Souza L , Dos‐Santos RC , et al. Acute ethanol modulates synaptic inhibition in the basolateral amygdala via rapid NLRP3 Inflammasome activation and regulates anxiety‐like behavior in rats. J Neurosci. 2023;43(47):7902‐7912. doi:10.1523/JNEUROSCI.1744-22.2023 37739795 PMC10669756

[28] Priyanka SH , Thushara AJ , Rauf AA , Indira M . Alcohol induced NLRP3 inflammasome activation in the brain of rats is attenuated by ATRA supplementation. Brain Behav Immun Health. 2020;2:100024. doi:10.1016/j.bbih.2019.100024 38377424 PMC8474578

[29] Zhang J , Hu T , Wang Y , et al. Investigating the neurotoxic impacts of arsenic and the neuroprotective effects of Dictyophora polysaccharide using SWATH‐MS‐based proteomics. Molecules. 2022;27(5):1‐21. doi:10.3390/molecules27051495 PMC8911851 35268596

[30] Zhang X , Yang H , Wang Y , et al. Proteomic study on the mechanism of arsenic neurotoxicity in the rat cerebral cortex and the protective mechanism of Dictyophora polysaccharides against arsenic neurotoxicity. ACS Chem Neurosci. 2023;14(12):2302‐2319. doi:10.1021/acschemneuro.3c00009 37272887

[31] Mohammadi R , Heidari MH , Sadeghi Y , Abdollahifar MA , Aghaei A . Evaluation of the spatial arrangement of Purkinje cells in ataxic rat's cerebellum after Sertoli cells transplantation. Folia Morphol (Warsz). 2018;77(2):194‐200. doi:10.5603/FM.a2017.0091 29064552

[32] Todd D , Clapp M , Dains P , Karacay B , Bonthius DJ . Purkinje cell‐specific deletion of CREB worsens alcohol‐induced cerebellar neuronal losses and motor deficits. Alcohol. 2022;101:27‐35. doi:10.1016/j.alcohol.2022.02.005 35378204 PMC9783827

[33] Bonthius DJ Jr , Winters Z , Karacay B , Bousquet SL , Bonthius DJ . Importance of genetics in fetal alcohol effects: null mutation of the nNOS gene worsens alcohol‐induced cerebellar neuronal losses and behavioral deficits. Neurotoxicology. 2015;46:60‐72. doi:10.1016/j.neuro.2014.11.009 25511929 PMC4339445

